# P-425. A Scoping Review of Data Systems for HPV Vaccination and HPV-Related Outcomes in China

**DOI:** 10.1093/ofid/ofae631.626

**Published:** 2025-01-29

**Authors:** Wei Wang, Ruixuan Wang, Ya-Ting Chen

**Affiliations:** Merck & Co., Rahway, New Jersey; Merck, Rahway, New Jersey; Merck, Rahway, New Jersey

## Abstract

**Background:**

Human papillomavirus (HPV) is a major cause of cervical cancer, the sixth most common cancer among women in China. In 2023, China launched a new government plan to facilitate the elimination of cervical, requiring reliable data systems for monitoring and evaluating progress. This study aimed to identify, understand, and summarize data systems that record HPV vaccination, cervical cancer screening, HPV infection, HPV-related cervical and non-cervical diseases, as well as sexual and life behaviors in China.

**Methods:**

A scoping review of literature from PubMed and the China National Knowledge Infrastructure databases was conducted, covering publications from 2012 to 2023. Two reviewers independently reviewed articles in two rounds, and all disagreements were resolved by a third reviewer.

**Results:**

From 234 studies, 146 data sources were identified, covering topics including HPV vaccination (n=2), HPV-related cervical and non-cervical diseases (n=121), cervical cancer screening and HPV infection (n=11), HPV-related knowledge, attitude, and belief (n=1), and sexual and life behaviors (n=7). The main source for HPV vaccination data is the National Immunization Program Information Management System, while HPV-related cancer data primarily come from the National Central Cancer Registry of China. Cervical screening and HPV infection data are recorded in clinical or laboratory databases, including KingMed Diagnostics Laboratory and the national “Two Cancer” Screening database. HPV knowledge, attitude and sexual behaviors are mainly collected from surveys of individuals registered in epidemiology registries. Notably, several cities, including Yinzhou, Chengdu, Suzhou, and Lecheng, have recently established population-level data systems linking vaccination, screening to cervical/other HPV outcomes; however, a national system linking HPV vaccination and cervical screening with HPV-related disease outcomes, health beliefs, and risk behaviors is lacking.

**Conclusion:**

There is a clear need for comprehensive and integrated data systems in China to effectively monitor and evaluate the impact and progress of public health intervention for cervical cancer elimination.

**Disclosures:**

**Wei Wang, PhD**, Merck: Stocks/Bonds (Public Company) **Ruixuan Wang, MPP**, Merck: Co-Op **Ya-Ting Chen, PhD**, Merck & Co., Inc: Stocks/Bonds (Private Company)

